# Laboratory method for investigating the influence of industrial process conditions on the emission of polycyclic aromatic hydrocarbons from carbonaceous materials

**DOI:** 10.1016/j.mex.2024.102687

**Published:** 2024-04-01

**Authors:** Katarina Jakovljevic, Thor Aarhaug, Heiko Gaertner, Kamilla Arnesen, Ida Kero, Gabriella Tranell

**Affiliations:** a NTNU, Alfred Getz’ vei 2B, Trondheim 7034, Norway; b SINTEF Industry, S P Andersens vei 3, Trondheim 7031, Norway; c SINTEF Industry, Alfred Getz’ vei 2B, Trondheim 7034, Norway

**Keywords:** PAHs emission, Carbon materials, PAH 16 & 42 analysis, Investigating the influence of industrial process conditions on the PAH emission from carbonaceous materials

## Abstract

This work is dedicated to developing a laboratory method for assessing emissions of polycyclic aromatic hydrocarbons (PAHs) from different carbon-based materials at elevated temperatures. The method will additionally contribute to enhancing the fundamental knowledge about the formation and decomposition of these compounds during various process conditions. Developing a method entails designing a setup for laboratory-scale experiments utilizing different furnace configurations and off-gas capturing media. To demonstrate the method's applicability, different carbon materials were tested under identical conditions, and analysis results for the same material in different furnace setups were compared. In this article, we have focused on the procedure for obtaining the “fingerprint” of PAH emissions under conditions characteristic of industrial processes.

• Two setups for investigation of the influence of temperature on PAH emissions were designed and tested for three types of carbon materials.
• The collected off-gas samples underwent analysis in two different laboratories to capture intra-laboratory differences and to evaluate the significance of the instrument detection limit.
• The results of PAH 16 (16 EPA PAH) and PAH 42 analysis were compared to showcase the influence of the expanded list on the overall emission of PAH.

The novel methodology enables the determination and comparison of PAH emissions during the thermal treatment of individual carbon materials under laboratory conditions. This could potentially be a new approach for predicting the PAH emissions in metallurgical industries that use these carbon materials as reducing agents in their processes and their control by optimizing process parameters and raw materials used. In addition to being suitable for simulating various conditions in the metallurgical industry, the utilization of low-hazard PAH solvents makes it a promising method.

## Graphical abstract

**Figure:**
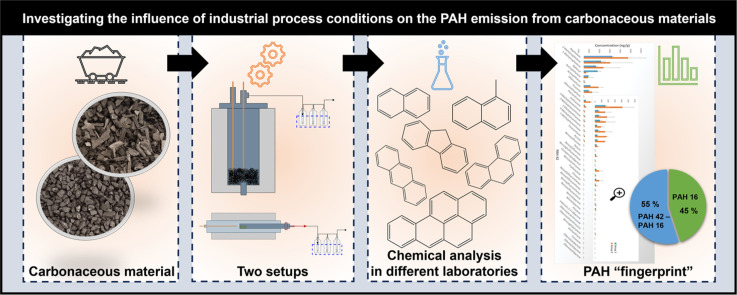

Specifications table

**Table:** 

Subject area:	Materials Science
More specific subject area:	Environmental Materials
Name of your method:	Investigating the influence of industrial process conditions on the PAH emission from carbonaceous materials
Name and reference of original method:	Laboratory method for investigating the influence of industrial process conditions on the emission of Polycyclic aromatic hydrocarbons from carbonaceous materials
Resource availability:	Entech 1400 vertical tube furnace, Nabertherm - RHTH 120–300/16–18 - Alumina tube furnace, Haake Fisons F3-CH Recirculating Heated Chiller Bath, Duran bubbler set with frit D0 (Paul Gothe GmbH)

## Background and state-of-art

Polycyclic aromatic hydrocarbons (PAHs) are a large class of organic compounds with two or more fused aromatic rings in their structural configurations. The most important sources of PAHs are incomplete combustion and pyrolysis of organic material, i.e., materials containing carbon and hydrogen.

In 1976, the United States Environmental Protection Agency (US EPA) named 16 PAHs as priority pollutants based on their toxicity and environmental presence in the highest concentrations [1]. Over the past 47 years, these 16 compounds have played an essential role as they have been analyzed in almost all types of environmental matrixes, which is why many regulations are focused on these group representatives. Out of hundreds of compounds in this group, the specific 16 compounds were selected based on the commercial availability of their analytical standards, the feasibility of measurement with available analytical methods, their occurrence in the environment, and, at that time, knowledge of their toxicity. However, despite the limited availability of data regarding the toxicological impact of polycyclic aromatic compounds beyond this list, research indicates that an extended list of polycyclic aromatic compounds should be used in the assessment of environmental pollution [[2], [3], [4], [5]]. For example, the list does not include substituted PAHs, such as alkylated ones, which are more abundant and persistent in the environment than the parent PAHs [[6], [7], [8]]. In addition, certain compounds, such as dibenzo pyrene isomers, exhibit a carcinogenic potential that is tenfold higher than benzo(a)pyrene [3], meaning that even small concentrations of these compounds can significantly contribute to the toxicity of an entire sample.

PAHs can be found throughout the environment in the air, water, food, and soil. The significance of air emissions lies in the fact that inhalation of PAH-containing air is considered the most common route of exposure to PAHs. Based on statistics provided by the Norwegian Environment Agency, it is evident that a considerable amount of polycyclic aromatic hydrocarbon (PAH) emissions in Norway for the year 2019 were released into the air [9]. Industrial activities, such as aluminum plants, manganese ferroalloy smelters, and silicon carbide producers, represent some of the largest sources of PAH emissions in Norway. Material producers use carbon materials as reductants, electrodes, etc., which results in varying degrees of emission of polycyclic aromatic hydrocarbons. These compounds can become airborne through many mechanisms. One such mechanism is evaporating PAH compounds that already exist in the materials. Another mechanism involves the thermal generation of PAH by incomplete combustion of carbon-containing material, whereby the emission of PAH represents a net of both the formation and decomposition of PAH compounds. In addition, mechanically generated dust particles can become airborne during the handling and transport of solid materials containing PAHs [10].

The current standard methodologies (e.g., ISO 11338–1) for determining PAH emissions from metallurgical industries often give inconsistent results, partly as a result of dynamic process conditions over shorter and longer time scales. Hence, measuring emissions with irregular intervals and short periods, although carried out according to standards and regulations, does not always help companies understand and control their emissions. Since the ultimate goal is to reduce the emission of these compounds, intensive work is presently carried out to develop new methods for online monitoring [11].

While industrial measurements are essential for emission reporting, no established, standard laboratory procedure exists for determining and comparing PAH emission from industrial carbonaceous materials under controlled conditions in laboratory-scale furnaces. The aim of the present work was, hence, to develop and evaluate a method for estimating PAH emission from different carbon materials in laboratory-scale experiments under various pyrolysis conditions. The new method should be applicable for determining the PAH emission under conditions similar to those of industrial processes, as well as being safe for the operator. As a basis for method development, the current study used temperatures and raw materials seen in the silicon (Si) and manganese (Mn) ferroalloy industries. Both metallurgical grade silicon (MG-Si) and manganese ferroalloys are produced by carbothermal reduction, most often in a Submerged Arc Furnace (SAF), with the use of both fossil and biological carbon materials as reductants, whereby, consequently, the resulting off-gas emissions may include varying amounts and types of PAHs [12,13].

## Method details

### Experimental laboratory setups

**Fig. 1:**
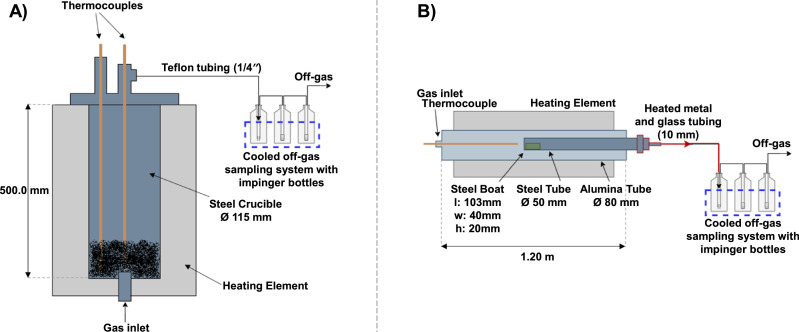
Setups used for PAH collection: (A) Setup 1 with Entech 1400 vertical furnace; (B) Setup 2 with Nabertherm - RHTH 120–300/16–18 – Horizontal Alumina tube furnace (AlTF1) [14].

To establish a successful laboratory methodology for evaluating PAH emission from different carbon materials under different conditions, it is necessary to design and test a suitable setup. In this work, two different setups (Fig. 1) were examined and compared to achieve the desired goal.

Fig. 1A shows Setup 1 using an Entech 1400 furnace. The furnace was fitted with a vertical crucible of a high-temperature resistant iron-chromium-aluminum alloy as a reaction chamber. The selected carbon material is heated while a carrier gas is injected through the bottom of the furnace. The reaction gases leave the crucible through a port in the top lid and are guided to the sampling equipment.

Fig. 1B shows Setup 2, which used a horizontal Nabertherm - RHTH 120–300/16–18 - Alumina tube furnace. In this furnace setup, the sample is placed in a steel holder at the very end of a steel tube, which is brought into the furnace from one end so that the sample is located in the middle of the alumina tube. The carbon material is heated while the carrier gas is injected from the opposite side of the steel tube inlet (left in the picture). The reaction gas leaves the furnace through a heated steel tube and goes to the sampling equipment.

Three washing bottles/bubblers/Impinger (250 mL Duran bubblers, one with Impinger nozzle and two with frit D0; with KS 19 pans and balls; Paul Gothe GmbH) are used to capture particulates and soluble constituents of the reaction gases in both setups. The first bottle is empty to prevent the washing solution from entering the furnace in case of reversed flow. Coarse particulates and condensate droplets will mainly be retained in the first washer, while the other two, filled with 2-propanol, will capture gaseous PAHs. During the sampling, these bottles were cooled to avoid the evaporation of the solvent and condensation of PAHs in the off-gas. In Setup 1, a cryostat bath with a water/glycol cooling system was used, while in Setup 2, an ice bath was used to which a new amount of ice was added periodically during the experiment.

### Test procedure

Before starting the experiment, the cryostat was turned on, and the temperature was set to −10 °C (Setup 1). While the cryostat reached the set temperature, a pre-set mass of representatively sampled carbon material was weighed and placed in the crucible (Setup 1) or the steel boat (Setup 2). The crucible/boat was then placed in the corresponding furnace and connected to the emission sampling system, ensuring no gas leakage.

**Fig. 2:**
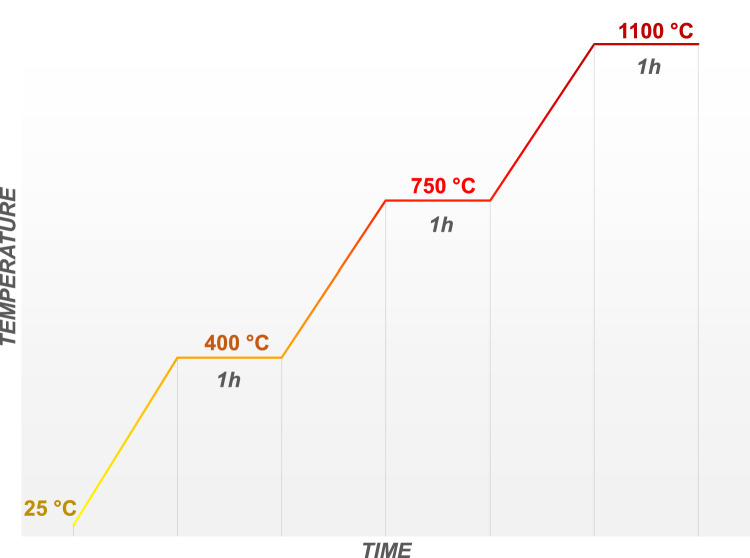
Temperature ramping diagram with three temperature set-points at which samples are taken.

During each experimental run, three sets of 3 washing bottles were used to capture PAHs released during three pre-set temperature ramping and holding phases: Phase I (room temperature to 400 °C), Phase II (401 to 750 °C), and Phase III (751 to 1100 °C), see Fig. 2. The sampling of PAH components during Phase 1 started with the temperature ramp from room temperature to 400 °C, with a ramp rate of 6 C° min^−1^ for Setup 1 and 5 C° min^−1^ for Setup 2, followed by 1 h holding time at 400 °C. After completing Phase 1, the set of bottles (Set#1) was collected, and the solvent and washing liquids were sent for analysis. Before the second temperature ramp was started, the set of washing bottles was replaced with Set#2, containing clean solvents, and the tube that connects the chamber with the bubbling bottles was washed with a small amount of fresh 2-propanol. The procedure was repeated for Phases II and III.

All carbon material samples used to perform the PAH emissions tests were weighed at room temperature before and after each experiment to investigate total sample mass loss.

### Sample analysis

The solvent samples were prepared and stored immediately after sampling. For each sampling sequence, the volume of 2-propanol from the two last bottles was combined with a small volume of fresh solvent used for washing all three bottles into one single fluid sample. The combined sample, ranging in volume from 220 mL to 280 mL (depending on the required amount of solvent for washing), was refrigerated in a glass bottle wrapped in aluminum foil (to avoid photolytic decomposition) at +4 °C until the chemical analysis was carried out.

The samples obtained from the experiments for method validation in Setup 1, with graphite as a carbon material, were analyzed for PAH 16 in the SINTEF Industry laboratory [15], as well as for PAH 42 (Table 1) in the NILU laboratory [16] which is accredited for those analyses, to compare the adequacy of the chemical analysis method for our samples, but also to determine the impact of the expanded list on the total amount of released PAHs.

**Table 1:** List of 42 PAH components.

Compound	MW (g mol^−1^)	*T_b_*^a^ ( °C)	# of rings
Naphthalene*	128.1	218	2
2-Methylnaphtalene	142.2	241	2
1-Methylnaphtalene	142.2	245	2
Biphenyl	154.2	255	2
Acenaphthylene*	152.2	279	3
Acenaphthene*	152.2	279	3
Dibenzofuran	168.2		3
Fluorene*	166.2	294	3
Phenanthrene*	178.2	338	3
Anthracene*	178.2	340	3
Dibenzothiophene	184.3		3
3-Methylphenanthrene	192.3	352	3
2-Methylphenanthrene	192.3	355	3
2-Methylanthracene	192.3	359	3
9-Methylphenanthrene	192.3	355	3
1-Methylphenanthrene	192.3	359	3
Retene	234.3		3
Fluoranthene*	202.3	383	4
Pyrene*	202.3	393	4
Benzo(*a*)fluorene	216.3	407	4
Benzo(*b*)fluorene	216.3	402	4
Benz(*a*)anthracene*	228.3	435	4
Triphenylene	228.3	439	4
Chrysene*	228.3	448	4
Benzo(*ghi*)fluoranthene	226.3	432	5
Cyclopenta(*cd*)pyrene	226.3		5
Benzo(*b*)fluoranthene*	252.3	481	5
Benzo(*k*)fluoranthene*	252.3	481	5
Benzo(*j*)fluoranthene	252.3	480	5
Benzo(*a*)fluoranthene	252.3		5
Benzo(*e*)pyrene	252.3	493	5
Benzo(*a*)pyrene*	252.3	496	5
Perylene	252.3		5
Dibenzo(*ac*)anthracene	278.3		5
Dibenzo(*ah*)anthracene*	278.3		5
Indeno(1,2,3-*cd*)pyrene*	276.3	536	6
Benzo(*ghi*)perylene*	276.3	550	6
Anthanthrene	276.3		6
Coronene	300.4	430	6
Dibenzo(*ae*)pyrene	302.4	414	6
Dibenzo(*ai*)pyrene	302.4		6
Dibenzo(*ah*)pyrene	302.4		6

⁎ US EPA PAH 16.

a PAH boiling point source [17], [18].

Identification and quantification of native PAHs in both laboratories were carried out using a gas chromatograph coupled to a mass spectrometer as a detector (GC/MS). All the samples were spiked with an internal standard containing deuterated PAH congeners. Details of the applied analysis methods are given in Table 2.

**Table 2:** Details of PAH analysis methods in two laboratories.

	SINTEF	NILU
Analysis	PAH 16	PAH 42
Instrument	GC/MSD (Agilent Technologies GC: 7890A with multimode inlet MSD: 5977A)	GC/LRMS (Agilent Technologies 79,800 GC coupled to an Agilent 5977 autosampler)
Analysis columns	Selected PAH, CP7462 30 m x 0.25 mm x 0.15 um ID	PAH Select
Injection principle	Direct injection, i.e., adds IS to the sample material, RT 54 min	Pulsed splitless
Syringe	10 µL	10 µL
Liners	5190–2293, splitless liner	Gooseneck splitless liner
Ion source	Xtr EI 350	EI+
Scan mode	Selected Ion Monitoring (SIM) mode	Selected Ion Monitoring (SIM) mode
Extraction Principle	Direct injection with/added IS	Liquid-liquid extraction - solvent exchange to cyclohexane. The extraction was followed with clean-up methods using “Grimmer method” followed by clean up using a deactivated silica column (adsorption chromatography).

The method detection limit (MDL) is defined as the minimum concentration of a substance that can be measured and reported with 99 % confidence that the value is above zero [19]. The MDL depends mainly on the instrument's sensitivity and the matrix effect. The MDL values in both laboratories for each compound from the PAH 16 and PAH 42 lists are summarized in **Table S1** in the Supplementary section.

### Cleaning procedure

A routine, seemingly simple cleaning operation is of great importance for this method if we bear in mind that for analytical analysis such as PAH 42 analysis, the equipment for performing experiments must be minimally contaminated before use. However, if we consider the type of material used, the cleaning operation is anything but simple.

In addition to glass bottles (bubblers), glass (Setup 2) or Teflon tubes (Setup 1), and small steel parts that connect the furnace and the bottles, the biggest challenge is cleaning the parts that are in direct contact with the carbon material.

The procedure for cleaning the steel crucible and its lid of Setup 1 involves first mechanical cleaning with a brush, then washing with acetone, and finally heating in a Muffle furnace to 650 °C in air and holding at temperature for 30–45 min in order to remove the remains of organic matter. The temperature of 650 °C was chosen following the temperature limitation of the available Muffle furnace of the appropriate size.

Cleaning the steel tube and the boat of Setup 2 involves washing with acetone and heating the tube furnace to 1300 °C in an atmosphere of synthetic air and keeping it at temperature for 30 min.

## Method validation

This work aims to develop a method to detect the specific composition of a PAH mixture as a unique signature or "fingerprint" in PAH compounds emitted from one particular carbonaceous material under controlled conditions. To validate the method's efficacy for acquiring a PAH emission fingerprint, we conducted experiments using various carbon materials and subsequently categorized the resulting data into three subsections. In the first one, we compared the analysis results of samples obtained in experiments with the same materials in different setups. Within this subsection, and in order to compare the efficiency of the designed setups, we also evaluated the efficiency of the cleaning procedure, the breakthrough test, and the repeatability of the conducted experiments. In the following subsection, we compared the methods of chemical analysis in two laboratories by comparing the analysis results of the same sample. Finally, we compared the total content of PAH 16 and PAH 42 and discussed the importance of the expanded list.

We did not examine the influence of the atmosphere on the PAH emission from carbonaceous materials for the purpose of method validation. However, using this approach, Arnesen et al. investigated the influence of the atmosphere on the emission of PAHs from green anode paste baking [14]. Nevertheless, a detailed examination of the impact of different atmospheres and flow conditions on the fingerprint of PAHs emitted from carbonaceous materials might be a potential subject for future work.

### Matrix and material

Three types of raw carbon materials (graphite, coke, and charcoal) were tested in Argon (Ar) atmosphere in the two different experimental setups, with three parallels for each experiment, resulting in 18 test runs, as shown in Table 3. Since three samples were collected for each experiment (for the three different temperature set-points), this resulted in 54 samples for PAH analysis.

**Table 3:** Experimental matrix.

	Setup 1	Setup 2
Graphite in Ar	3 parallels	3 parallels
Coke in Ar	3 parallels	3 parallels
Charcoal in Ar	3 parallels	3 parallels

The coke and charcoal, materials for the experiments, were provided by an industrial partner, and the chemical analysis of these materials is presented in **Table S2** in the Supplementary section.

In addition to coke and charcoal, graphite grade G330 (Schunk Tokai) was used as a reference material. The total ash content in this material is ≤ 300 ppm, according to property information obtained from the supplier. Graphite, the most stable form of carbon, was chosen for similar reasons as Argon, as, under ideal conditions, it should contain nothing but carbon. The effect of temperature on pure carbon in an inert atmosphere could hence be used as a baseline to compare other carbon materials.

Argon gas purity grade 5.0 was chosen for the atmosphere to test the efficiency of the setup, the difference between the raw materials, and the influence of temperature, initially eliminating the additional influence of the atmosphere to establish a reliable baseline for future studies in different atmospheres.

Isopropyl alcohol (≥ 98 % Technical, VWR Chemicals) was used as a suitable solvent for capturing polycyclic aromatic hydrocarbons. PAHs are lipophilic non-polar compounds; however, substituted groups can contribute to their polarity [20]. These compounds exhibit low solubility or insolubility in water but are soluble in organic compounds such as toluene, benzene, carbon tetrachloride, etc. Isopropanol is, in addition to being less toxic than, for example, toluene, also a better choice than, for example, acetone, which has a low boiling point.

Preparation of the carbon material samples included sieving to specific particle size (5–10 mm for graphite and coke and 5–25 mm for charcoal), making a representative sample according to the literature procedure (Spoon method) [21], and drying at 107 °C ± 3 °C to constant mass [22], in an Entech muffle furnace in air. The sample's initial weight was measured before the experiment: for Setup 1, the materials weighed 300 g, while for Setup 2, the graphite and coke weighed 15 g, and the charcoal weighed 10 g.

### Comparing setup 1 and setup 2

Conducting parallel experiments using the same materials, temperatures, and atmosphere in both setups serves to evaluate the setups in terms of usability and simultaneously compare the scientific outcomes of the experiments.

The most significant differences between the two setups are the amount and position of the sample, and the orientation of the gas flow. While the crucible in Setup 1 is 50 cm high and has an internal diameter of 11.5 cm, the sample holder in Setup 2 has dimensions of 10.3 cm x 4 cm x 2 cm, which significantly limits the amount of sample to be tested, especially for the materials with low relative density such as charcoal. However, if the desired goal can be achieved by using a smaller sample, this "limitation" does not have to be a disadvantage, as working with smaller samples is easier and safer for the operator. While Setup 1 has a vertical crucible, in which the carrier gas enters from the bottom and passes evenly through the sample, Setup 2 has a horizontal steel tube in which the boat with the sample is placed, and the carrier gas comes from the side, which means that it does not pass through the sample but moves above it. In the presented experiments, inert gas was used, so the advantages and disadvantages of this difference cannot be fully determined from a gas-solid reaction point of view.

**Fig. 3:**
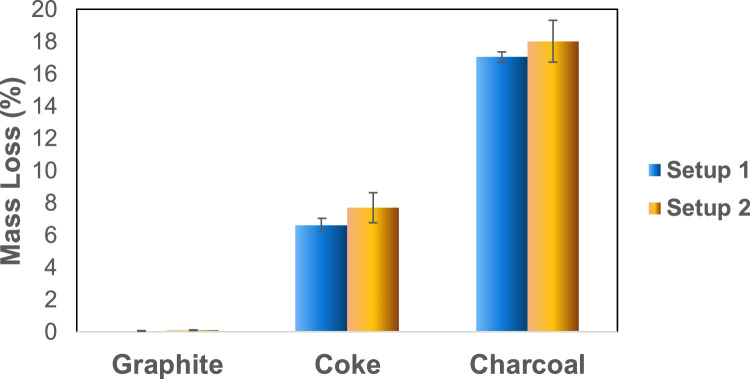
Average total mass loss (%) over the temperature cycle for carbon material samples in both setups. Error bars show the variation in triplicate experiments.

As mentioned above, all carbon material samples were weighed at room temperature before and after the experiment. Fig. 3 shows the average mass loss (%) for each carbon material used in the experiments in both setups. Most of the mass loss may be caused by the thermal evaporation of volatile organic components in the sample and is very similar in both setups for each material, although slightly lower values (up to 1 % of total loss) were observed in the experiments in Setup 1.

Table 4 presents the results of the PAH 42 analysis as total PAHs emitted from carbon materials tested in both setups. While for graphite, the difference in total PAHs between setups is almost insignificant, the value for charcoal in Setup 2 is nearly double the value for Setup 1.

**Table 4:** Total PAH emitted from carbon materials in Setup 1 and Setup 2. Values represent mean of two parallels for graphite in Setup 1 and means of three parallels for all other experiments.

	Concentration (µg g^−1^)	
	Setup 1	Setup 2
Graphite	2.4	2.0
Coke	17.8	26.4
Charcoal	49.9	99.2

**Fig. 4:**
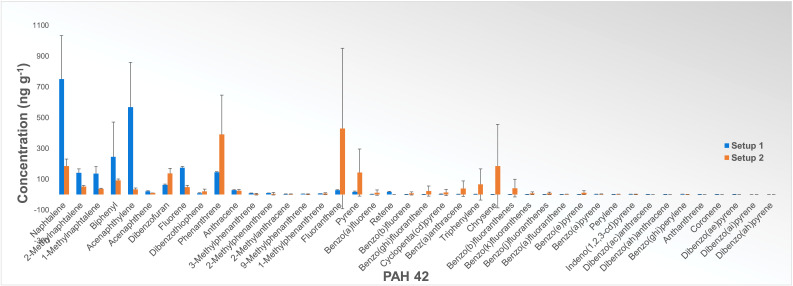
Results of PAH 42 analysis (NILU) in ng g^−1^ sample reacted for experiments with graphite as a carbon material performed in Setup 1 (blue) and Setup 2 (orange). Results are the total amount of each PAH for the full temperature range. Error bars show the standard deviation for each compound based on two parallel experiments in Setup 1 and three parallel experiments in Setup 2.

Fig. 4 shows the results of PAH 42 analysis for experiments with graphite as a carbon material in an Argon atmosphere, measured as total PAH across the full temperature range in ng g^−1^ sample reacted. Since graphite is the most stable form of pure carbon and Argon is an inert gas, the release of PAHs should be limited. However, the results show PAH emission even under these conditions, although the amount of released PAH compounds is very low in both setups. Although the difference in total PAH between the results for the two setups is even minor compared to other tested materials, we can see a significant discrepancy between the compound concentration trends. The differences in the compound concentration trends between the two setups led us to examine the influence of furnace contamination on the analysis results, which will be discussed later in the “Validation of cleaning procedure” section.

**Fig. 5:**
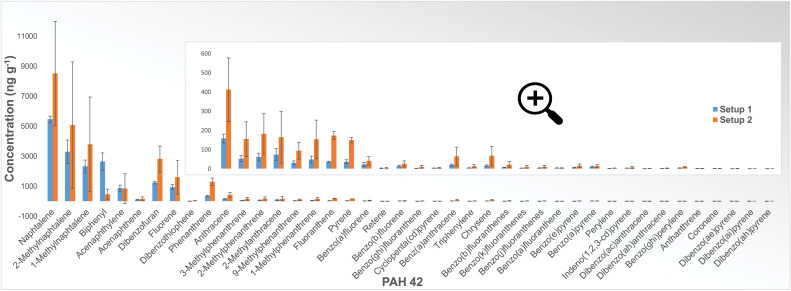
Results of PAH 42 analysis (NILU) in ng g^−1^ sample reacted for experiments with coke as a carbon material performed in Setup 1 (blue) and Setup 2 (orange). Results are the total amount of each PAH for the full temperature range. Error bars show the standard deviation for each compound based on three parallel experiments.

If we compare the results of PAH 42 analysis of samples obtained from experiments with coke as a carbon material performed in two setups (Fig. 5), we can see that despite differences in absolute concentration, the distribution trend is very similar for all compounds except for a few (e.g., Biphenyl, 2-Methylanthracene, Fluoranthene).

**Fig. 6:**
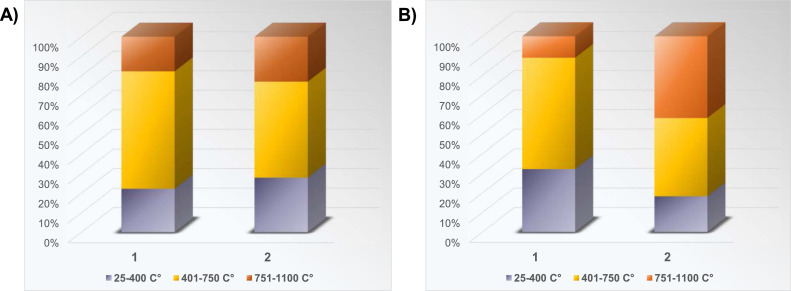
Distribution of LMW and HMW PAHs released in experiments with coke in: (A) Setup 1, and (B) Setup 2, with temperature.

For a convenient presentation of the results, 42 compounds are divided into two groups, one which includes compounds with 2 and 3 rings - Low Molecular Weight PAHs (LMW PAHs), and the other which includes other compounds with 4–6 rings - High Molecular Weight PAHs (HMW PAHs). Fig. 6 shows that most LMW and HMW PAHs were released in Phase II of the experiment with coke in Setup 1, i.e., at a temperature of 401–750 °C. The same is the case for the experiment with coke in Setup 2, although a large proportion of the released HMW PAHs is also in Phase III of the experiment, i.e., at temperatures of 751–1100 °C. Although the boiling points of LMW PAHs range from 218 °C for Naphthalene to 390 °C for Retene, and the boiling points of HMW PAHs from 383 °C for Fluoranthene to 550 °C for Dibenzo(ah)anthracene, it can be seen from the results that most of these compounds are released at temperatures higher than their boiling points. The experimental results suggested that PAHs emitted during the pyrolysis process at low temperatures mainly came from evaporation of the aromatic structures initially within the coke. With a further increase in pyrolysis temperature, PAH production initially increases, giving a maximum peak in Phase II of the experiment, after which it decreases with increasing temperature, indicating that two competitive reactions occur during coke pyrolysis: PAH formation and PAH decomposition. It was postulated that at temperatures below 750 °C the dominant reaction is the PAH formation. One possible mechanism of their formation via pyrosynthesis from low hydrocarbons is that higher pyrolysis temperature (> 500  °C) breaks carbon–hydrogen, and carbon–carbon bonds to form free radicals, which will combine with acetylene, followed by their further condensation with aromatic ring structures [23]. This aromatization process might give off polycyclic aromatic hydrocarbons in Phase II of the experiments with coke in both setups. Similar behavior was observed with PAH production from coal pyrolysis, where PAH emission concentrations reached the maximum at a pyrolysis temperature of 800 °C [24].

**Fig. 7:**
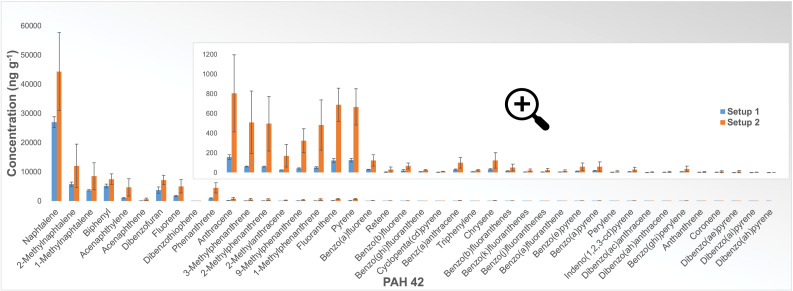
Results of PAH 42 analysis (NILU) for samples obtained from experiments with charcoal as a carbon material performed in Setup 1 (blue) and Setup 2 (orange). Error bars show the standard deviation for each compound based on three experiments.

Almost the same can be observed by comparing the results of the analysis of samples obtained by experiments with charcoal in the two setups (Fig. 7). Again, with a few exceptions (Biphenyl, Coronene), the distribution trend is very similar in both setups.

**Fig. 8:**
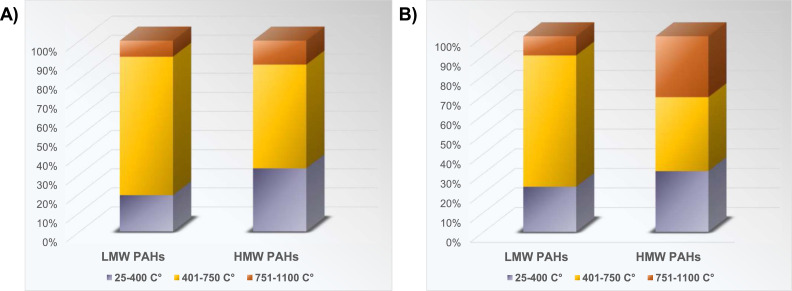
Distribution of LMW and HMW PAHs released in experiments with charcoal in (A) Setup 1, and (B) Setup 2, with temperature.

In experiments with charcoal, in both setups, the most significant amount of both LMW and HMW PAHs was released in Phase II, that is, at temperatures of 401–750 °C. However, as in the previous case, the trend is somewhat different for HMW PAHs since a large amount of these compounds was also released in Phase III of the experiment in Setup 2 (Fig. 8).

### Validation of cleaning procedure

**Fig. 9:**
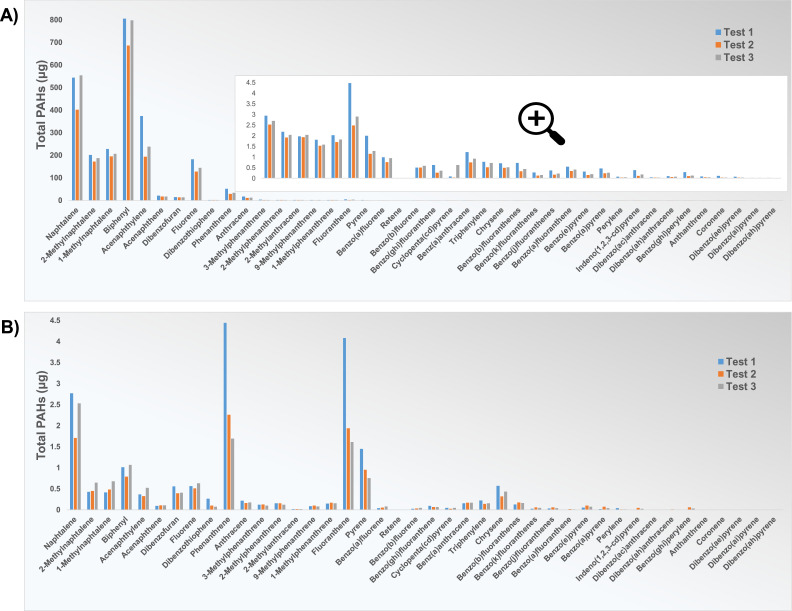
Results of PAH 42 analysis (NILU) for triplicate experiments in empty furnaces in an atmosphere of Ar in (A) Setup 1, and (B) Setup 2.

To determine the cleaning efficiency of both setups, three consecutive tests, to ensure no other material was introduced into the furnace between tests, were performed with no carbon sample in the furnace, with a standard cleaning procedure before the first and between each test. The experiments were performed in an Argon atmosphere, like those with carbon materials. The only difference was that the bubbling bottles were not changed after each temperature phase, but one sample was taken at the end of the experiment. The results are shown in Fig. 9 and represent the amounts of released PAHs due to the contamination of the crucible/sample holder.

From the results shown in Fig. 9, it can be concluded that there is some contamination of the sample holders, even after multiple cleanings. Although contamination was highest in test 1, neither performing multiple cleaning cycles nor additional thermal treatment in the form of a subsequent experiment contributed significantly to the reduction of contamination, that is, to the reduction of the amount of PAHs released from the empty furnaces.

**Fig. 10:**
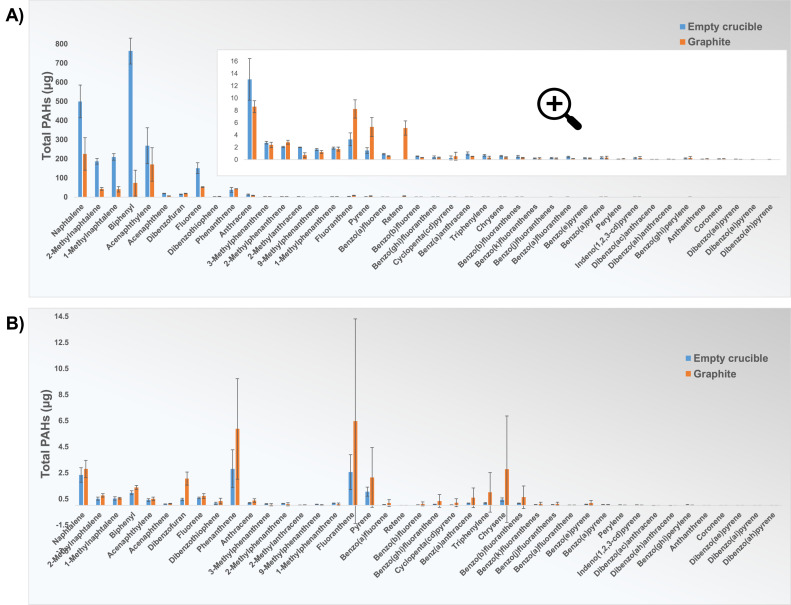
Results of PAH 42 analysis (NILU) for experiments in empty furnaces (blue) and experiments with graphite as a carbon material (orange), in an atmosphere of Ar in (A) Setup 1, and (B) Setup 2. Error bars show the standard deviation for each compound based on three parallel experiments, except for experiments with graphite in Setup 1, where error bars show standard deviation based on two parallel experiments.

Fig. 10 shows a comparison of the amount of released PAH compounds from experiments with no material and those with graphite as carbon material. Apart from the trend being quite similar, the concentrations of released compounds from empty furnaces are also significant compared with those from experiments with graphite, indicating that the influence of contamination is considerable when studying a material with very low PAH emission. Most of the released PAHs in the graphite experiments could hence be due to contamination of the furnace. From the test results for Setup 1, even higher concentrations of many PAH compounds can be observed in the empty furnace, so the amount of total PAHs is almost three times higher than one using graphite (Table 5), which may depend on the type of material used in the previous experiments. In addition, compared to the sample holder of Setup 2, the crucible of Setup 1 has a significantly larger surface area relative to the sample size; therefore, greater contamination can be expected in this setup.

**Table 5:** Total PAH emitted from experiments with an empty furnace and experiments with graphite, performed in both setups. The values indicate the average of two tests conducted for graphite in Setup 1, while for all other experiments, the average is calculated from three tests.

	Total PAH (µg)	
	Empty furnace	Graphite
Setup 1	2188.9	717.3
Setup 2	14.5	30.5

The traces of materials used in these furnaces are, given the thorough furnace cleaning procedure, clearly not easily removed by solvents that are entirely or relatively safe for use, and the thermal profile and holding time of thermal treatment in the air need further adjustment to completely remove contamination traces. Since the method of analysis of polycyclic aromatic hydrocarbons is sensitive, detecting these compounds as a consequence of contamination is inevitable if present. Although the concentrations of the detected compounds are not high enough to significantly affect the fingerprint of other materials than graphite, for precise analysis, this contamination should be taken into account and be considered in error calculation or as a baseline if it cannot be avoided. There is no standardized methodology for procedural blanks in the scientific literature. However, the concept and importance of procedural blanks, which assess contamination throughout the measurement process, are widely recognized in terms of their impact on the reliability of produced data [25,26]. Therefore, we suggest that an empty furnace trial is always run prior to measurement to determine the level of baseline emission from the setup.

### Breakthrough test

To test the efficiency of the sampling line, i.e., the breakthrough of PAHs through the third bubbling bottle, two additional experiments were performed with charcoal in an Argon atmosphere in Setup 2 (the same sampling line was used in both setups). Breakthrough testing was performed by adding an analytical thermal desorption (ATD) tube filled with Tenax TA adsorbent and glass wool to the sampling line, behind the last bubbling bottle. If there is a breakthrough, off-gas from the furnace will pass through three bubbling bottles, the first of which is empty, the following two filled with approximately 100 mL of 2-propanol, and the ATD tube, where in this case, the LMW PAH will be absorbed. The role of the glass wool was to shield the absorbent material by filtering any particles in the stream. The PAH content in the ATD tubes was analyzed using thermal desorption and GC–MS. Table 6 shows the results of PAHs in the tubes, compared to the mean of three experiments of PAHs in 2-propanol.

**Table 6:** Concentrations of PAH species detected in tubes and 2-propanol after the experiment with charcoal in Argon atmosphere. Values in 2-propanol are an average of three experiments, and results from tubes are the average of two experiments.

PAH Specie	Tube (µg g^−1^)	2-propanol (µg g^−1^)
Naphthalene	0.00	44.35
Acenaphthene	0.00	0.59
Phenanthrene	0.40	4.50
Anthracene	0.63	0.80
Fluoranthene	0.82	0.69
Triphenylene	1.61	0.02

The results show the presence of negligible concentrations of the lightest compounds, but a significantly higher concentration of Fluoranthene and Triphenylene can be observed. The solubility of Naphthalene and Acenaphthene in 2-propanol is higher than that of the remaining listed compounds [27], which could explain the observed breakthrough. Alternatively, the higher concentration of Fluoranthene and Triphenylene might be attributed to the evaporation of 2-propanol containing PAH from the sampling line to the tube if the solvent is not kept at a sufficiently low temperature since, in these two experiments, a loss of 4.04–6.14 mL of 2-propanol was observed for each temperature set point, i.e., an average loss of 2.30 % of 2-propanol per experiment. A similar occurrence was observed by Arnesen et al., who tested the breakthrough of LMW PAHs from 2-propanol into the sample collection system to investigate the reason for the small amount of LMW PAHs for all experiments performed compared to HMW PAHs [14]. Nevertheless, the influence of solvent evaporation is improbable as it would also result in elevated levels of the lightest compounds in the tube.

As mentioned above, in Setup 1, a cryostat bath with a water/glycol cooling system was used for cooling 2-propanol, and the temperature of the bath was maintained at −8 to - 10 °C, while in Setup 2, an ice bath was used, which makes controlling the temperature of the solvent more difficult. However, a slight evaporation of 2-propanol was also observed in Setup 1. As such, we suggest using a cryostat for more reliable low-temperature cooling to ensure minimum solvent evaporation.

### Repeatability of measurements

Observing the error bars in Fig. 5, Fig. 7, we can conclude that the quantitative repeatability of the experiments is low, particularly for Setup 2. The coefficients of variation from the mean values of the total amount of PAHs per tested materials are shown in Table 7.

**Table 7:** Coefficient of variation (CV) for all sets of PAH 42 analysis results (NILU) for each individual carbon material in both setups. CV was calculated using the equation: CV (%) = SD/ x̅ * 100 %, where: SD = standard deviation, and x̅ = mean of three measurements.

	Coefficient of Variation (%)	
	Setup 1	Setup 2
Graphite	31.03	83.14
Coke	4.88	53.05
Charcoal	8.08	38.89

Based on the displayed coefficients of variation, we concluded that we had better experimental control in Setup 1. The possible reason for this could be the contamination of the furnace since, between sets of three tests, the furnace of Setup 2 was used for other experiments. Also, one of the reasons could be the contamination of the alumina tube due to the high-temperature gradient in the setup, causing the diffusion of condensate on the walls [28], and the impossibility of its complete extraction through the steel tube into the sampling system. Despite this, the trend of concentrations of released PAH compounds for all materials is comparable for the setups, providing insight into the PAH emission fingerprint from carbonaceous materials, which is the objective of this method.

Other advantages and disadvantages of both setups should be considered, such as controlling the temperature, the time required to start the experiment, i.e., assembling the setup, the efficiency and the time it is necessary to spend for cleaning, as well as the required amount of chemicals, etc. In the context of temperature control, it is not uncommon for the actual temperature in furnaces, such as those employed in this study, to exhibit deviations from the desired set temperature. Therefore, the minor deviation between the observed temperature and the predetermined temperature, as well as the slight variation in temperature over parallels in Setup 2, can be regarded as advantageous features of this setup compared to Setup 1.

### Comparing results of PAH analysis in different laboratories

Although this work was primarily focused on developing a laboratory method for comparing emission of PAHs from different carbon materials under different conditions such as temperature and atmosphere, consistent and accurate analysis of the samples obtained from the experiments is clearly of great importance for the results. Selection of a laboratory for sample analysis is based on several criteria such as possession of appropriate standards and developed methodology for PAH analysis, instrument sensitivity, as well as the price and time required for analysis.

**Fig. 11:**
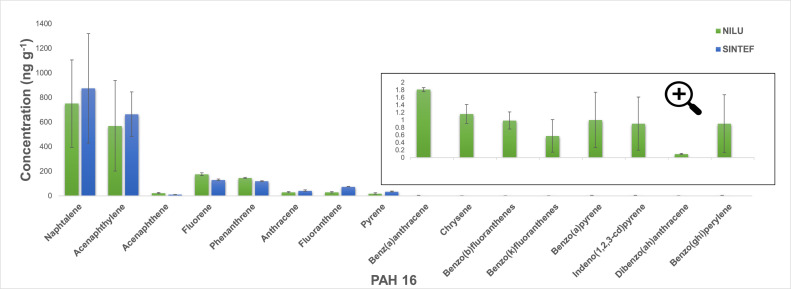
Results of PAH 16 analysis performed at NILU (green) and SINTEF (blue) laboratories for samples obtained in experiments (Exp1 and Exp 2) with graphite as a carbon material in Setup 1. Values represent the total amount of PAH emitted over the 25–1100 °C temperature range per gram of graphite sample. Error bars show the standard deviation for each compound based on two experiments.

As described under the Method details, the first set of samples obtained from the experiments in Setup 1, with graphite as the carbon material, were primarily analyzed for PAH 16 at the SINTEF laboratory in Trondheim. However, the results showed that only 8 out of 16 PAHs were detected (Naphthalene, Acenaphthylene, Acenaphthene, Fluorene, Phenanthrene, Anthracene, Fluoranthene, Pyrene), with Naphthalene accounting for 39–54 % of the total amount of PAHs in the samples. In contrast, some higher molecular weight PAHs (Benz(a)anthracene, Chrysene, Benzo(b)fluoranthenes, Benzo(k)fluoranthenes, Benzo(a)pyrene, Indeno(1,2,3-cd)pyrene, Dibenzo(ah)anthracene, Benzo(ghi)perylene) were not detected in any sample, indicating that their concentration was below the lower detection limit (LDL) of the instrument, which can be seen in Fig. 11 (blue bars).

**Fig. 12:**
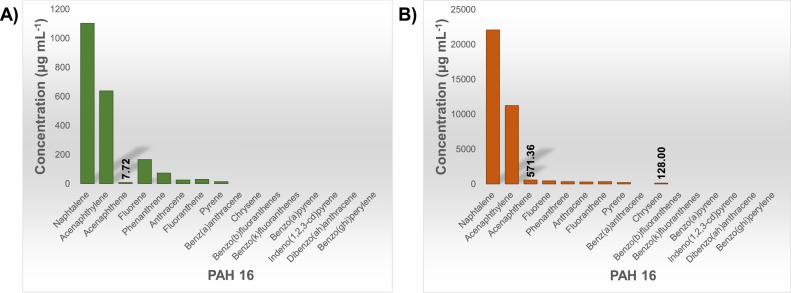
Results of PAH 16 analysis (SINTEF): (A) before, and (B) after concentrating sample obtained in experiment with graphite as a carbon material (Exp3) performed in Setup 1.

As a low PAH concentration was expected in samples from the reaction of graphite, samples obtained in one experiment (Exp3) were pre-concentrated and reanalyzed. As expected, the results after concentration showed a significantly higher concentration of the previously detected compounds and also detected Chrysene. The other seven compounds from the PAH 16 list were, however, still not detected (Fig. 12).

Samples obtained in two of the three parallel experiments were subsequently analyzed at the NILU laboratory in Kjeller. Comparing the analysis results from the different laboratories, it can be observed that the concentrations of some compounds (especially low molecular weight species such as Naphthalene and Acenaphthylene) are higher in SINTEF's results, resulting in higher total PAH values. The mean values of total PAHs calculated from SINTEF's and NILU's results are 1.9 µg g^−1^ and 1.7 µg g^−1^, respectively. In contrast to the SINTEF's analysis, all 16 compounds were detected in NILU's analysis (Fig. 11). On the other hand, if we compare the concentrations of the compounds detected in both laboratories and their distribution with temperature, the trend is similar. Many of the samples used in the current study required a very low detection limit in the analysis, and hence, all further samples were analyzed at the NILU laboratory.

### Comparing PAH 16 and PAH 42

**Fig. 13:**
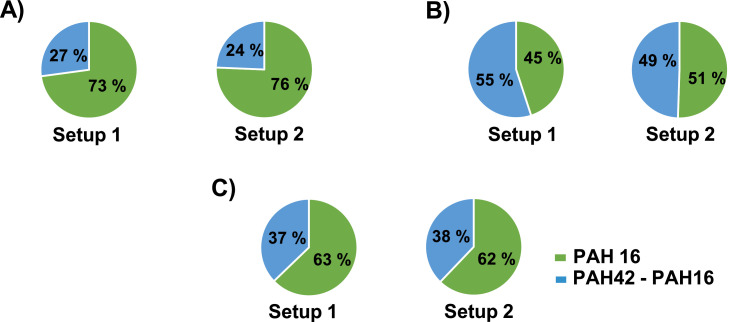
PAH 16/PAH 42 ratio (average values of the sum of three tests) for: (A) graphite, (B) coke, and (C) charcoal.

Fig. 13 presents the ratio of PAH 16 and PAH 42 for all tested carbon materials, where the mean value of the three measurements for each experiment are shown. It can be seen from the results that PAH 16 makes up 45–76 % of the total amount, which implies that the remaining compounds can make up more than 50 %, which is the case for the results of the analysis of the samples obtained by the thermal treatment of coke. Of these, 12–34 % are 1-Methylnaphthalene and 2-Methylnaphthalene, compounds that are known to be more persistent than the parent compound, Naphthalene. In addition, although limited, data on the toxicity of these compounds can be found in the literature [[29], [30], [31]].

Given the discussion above regarding the contamination of the furnace, it is crucial to consider how this contamination can impact the analysis results. Specifically, when examining the PAH16/PAH42 ratio in graphite, it becomes evident that furnace contamination has a greater influence compared to other materials due to the minimal emission levels associated with graphite. Consequently, it is important to acknowledge that the results for graphite carry a larger margin of error.

## Summary

• Two experimental setups were designed and tested to develop a method for studying how different process conditions affect the emission of PAH from carbon materials. Three carbon materials (graphite, coke, and charcoal) were tested in an inert atmosphere (Ar).
• A comparison was made between the PAH analysis methods used in two different laboratories. In the SINTEF laboratory, eight out of sixteen PAH 16 compounds were detected in all analyzed samples. The method detection limit plays an essential role in the PAH analysis of samples obtained by thermal treatment of carbon materials. For our samples, the more suitable sensitivity of the instrument was found in the NILU laboratory, although both methods proved to be suitable for PAH analysis.
• All samples were analyzed for PAH 42 instead of PAH 16, and it was shown that PAH compounds of the extended part of the list make up a significant proportion of the total amount of released PAHs, with 49–55 % and 37–38 % for coke and charcoal, respectively.
• Regarding the influence of temperature, the highest emission of PAHs for both coke and charcoal was observed in the second phase of the experiment, that is, at a temperature of 401–750 °C.
• Comparing the results of PAH 42 analysis of samples obtained from experiments in two setups, it can be concluded that, despite challenges with cleaning, the distribution trend is very similar for all compounds except for a few, indicating that both setups are applicable to achieve our goal, the PAH emission fingerprint.

## Limitations and future work

From the results of the method validation experiments, we observed that better experimental control was achieved in experiments in Setup 1, with a coefficient of variation ranging from 2.83 to 30.44. However, to use this method to obtain more precise emission data on PAH emissions from carbon materials, it is important to consider contamination.

In addition to the need for more extensive research on cleaning methods, there are various opportunities for future research that would enhance the capabilities of the developed method to acquire an even more distinct pattern of PAH emissions from carbonaceous materials. Investigating the impact of various atmospheres and flow rates on PAH emissions during the thermal treatment of carbon materials could provide a comprehensive understanding of the method's applicability for quantitatively determining PAH emission fingerprints in conditions like those in industrial activities.

## Ethics statements

This work did not involve human subjects, animal experiments data, and data collected from social media platforms.

## CRediT authorship contribution statement

**Katarina Jakovljevic:** Investigation, Writing – original draft, Formal analysis. **Thor Aarhaug:** Conceptualization, Formal analysis. **Heiko Gaertner:** Methodology. **Kamilla Arnesen:** Methodology. **Ida Kero:** Conceptualization, Project administration. **Gabriella Tranell:** Supervision, Conceptualization, Writing – review & editing.

## Declaration of competing interest

The authors declare that they have no known competing financial interests or personal relationships that could have appeared to influence the work reported in this paper.

## Data Availability

Data will be made available on request.

## Appendix Supplementary materials

Image, application 1
