# Supplementary material for: Laboratory method for investigating the influence of industrial process conditions on the emission of polycyclic aromatic hydrocarbons from carbonaceous materials
Source: MethodsX. 2024 Apr 1;12:102687. doi: 10.1016/j.mex.2024.102687 (PMC11015522; doi:10.1016/j.mex.2024.102687)
Supplement: Supplementary file 1 [file mmc1.docx]

**SUPPLEMENTARY MATERIAL**

**Laboratory method for investigating the influence of industrial process conditions on the emission of Polycyclic aromatic hydrocarbons from carbonaceous materials**

Katarina Jakovljevic, Thor Aarhaug, Heiko Gaertner, Kamilla Arnesen, Ida Kero, Gabriella Tranell

INDEX:

**Table S 1** Method detection limit (MDL) values for PAH compounds in two laboratories.^a^ The units are expressed as the mass (ng) of the PAH compound per one milliliter of solution (in our case, isopropanol solution).

**Table S 2** Chemical analysis of raw materials used in experiments.^a^

**Table S 1** Method detection limit (MDL) values for PAH compounds in two laboratories. The units are expressed as the mass of the PAH compound per one milliliter of solution (in our case, isopropanol solution).

|  | **MDL** | |
| --- | --- | --- |
| **Compound** | **SINTEF^a^ (μg/ml)** | **NILU^b^ (ng/ml)** |
| Naphthalene* | 0.08 | 0.821 |
| 2-Methylnaphtalene |  | 0.387 |
| 1-Methylnaphtalene |  | 0.200 |
| Biphenyl |  | 0.238 |
| Acenaphthylene* | 0.16 | 0.088 |
| Acenaphthene* | 0.09 | 0.225 |
| Dibenzofuran |  | 0.368 |
| Fluorene* | 0.25 | 0.481 |
| Phenanthrene* | 0.01 | 1.350 |
| Anthracene* | 0.01 | 0.030 |
| Dibenzothiophene |  | 0.041 |
| 3-Methylphenanthrene |  | 0.296 |
| 2-Methylphenanthrene |  | 0.384 |
| 2-Methylanthracene |  | 0.015 |
| 9-Methylphenanthrene |  | 0.199 |
| 1-Methylphenanthrene |  | 0.209 |
| Retene |  | 0.843 |
| Fluoranthene* | 0.18 | 0.452 |
| Pyrene* | 0.01 | 0.014 |
| Benzo(*a*)fluorene |  | 1.410 |
| Benzo(*b*)fluorene |  | 0.010 |
| Benz(*a*)anthracene* | 0.01 | 0.051 |
| Triphenylene |  | 0.011 |
| Chrysene* | 0.05 | 0.045 |
| Benzo(*ghi*)fluoranthene |  | 0.054 |
| Cyclopenta(*cd*)pyrene |  | 0.092 |
| Benzo(*b*)fluoranthene* | 2.00 | 0.064 |
| Benzo(*k*)fluoranthene* | 1.01 | 0.014 |
| Benzo(*j*)fluoranthene |  | 0.016 |
| Benzo(*a*)fluoranthene |  | 0.006 |
| Benzo(*e*)pyrene |  | 0.039 |
| Benzo(*a*)pyrene* | 1.00 | 0.007 |
| Perylene |  | 0.006 |
| Dibenzo(*ac*)anthracene |  | 0.012 |
| Dibenzo(*ah*)anthracene* | 20.00 | 0.018 |
| Indeno(1,2,3-*cd*)pyrene* | 10.00 | 0.017 |
| Benzo(*ghi*)perylene* | 2.00 | 0.013 |
| Anthanthrene |  | 0.013 |
| Coronene |  | 0.027 |
| Dibenzo(*ae*)pyrene |  | 0.034 |
| Dibenzo(*ai*)pyrene |  | 0.034 |
| Dibenzo(*ah*)pyrene |  | 0.037 |
| *US EPA PAH 16  ^a^A. Brunsvik, “Private communication.” Dec. 21, 2023. | | |
| ^b^A. K. Halse, “Private communication.” Sep. 16, 2022. | | |

**Table S 2** Chemical analysis of raw materials used in experiments.^a^

|  | **Value (%)** | |
| --- | --- | --- |
|  | **Coke** | **Charcoal** |
| Moisture | 13.53 | 3,40 |
| Fix C (DB) | 91.97 | 85.2 |
| Fix C (WB) | 79.52 | 82.3 |
| Ash (DB) | 2.04 | 2.1 |
| Ash (WB) | 1.93 | 2 |
| Volatiles (DB) | 5.99 | 12.7 |
| Volatiles (WB) | 5.66 | 12.3 |
| P (DB) | 0.186 |  |
| Fe/100FC | 0.14 | 0.019 |
| Al/100FC | 0.24 | 0.0313 |
| Ca/100FC | 0.06 | 0.6591 |
| Ti/100FC | 0.01 | 0.0015 |
| Mg/100FC | 0.04 | 0.0803 |
| Na/100FC | 0.038 |  |
| K/100FC | 0.01 |  |
| P/100FC | 0.004 | 0.0689 |
| Si/100FC | 0.57 |  |
| Fe_2_O_3_ | 8.83 | 1.1 |
| SiO_2_ | 55.06 | 23.4 |
| Al_2_O_3_ | 20.03 | 2.4 |
| CaO | 3.63 | 37.4 |
| TiO_2_ | 0.9 | 0.1 |
| MgO | 3.09 | 5.4 |
| Na_2_O | 2.296 | 2.8 |
| K_2_O | 0.566 | 5.6 |
| SO_3_ | 3.55 | 2.5 |
| MnO | 0.12 | 2.8 |
| P_2_O_5_ | 0.425 | 6.4 |
| Cr_2_O_3_ | 0.019 |  |
| ^a^ Myrvågnes V. “Private communication.” Jan. 10, 2020. | | |
